# The impact of Post-COVID-Syndrome on functioning – results from a community survey in patients after mild and moderate SARS-CoV-2-infections in Germany

**DOI:** 10.1186/s12995-021-00337-9

**Published:** 2021-10-07

**Authors:** Christina Lemhöfer, Christian Sturm, Dana Loudovici-Krug, Norman Best, Christoph Gutenbrunner

**Affiliations:** 1 Department of Physiotherapy, Jena University Hospital, Jena, Germany; 2 Department of Rehabilitation Medicine, Hannover Medical School, Hannover, Germany

**Keywords:** Long-COVID, Mild and moderate COVID-19, Impact on functioning, Quality of life

## Abstract

**Background:**

In COVID-19 survivors a relatively high number of long-term symptoms have been observed. Besides impact on quality of life, these symptoms (*now called Post-COVID-Syndrome*) may have an impact on functioning and may also hinder to participation in social life in affected people. However, little is known about developing such syndrome a for patients with mild and moderate COVID-19 who did not need hospitalization or intensive care.

**Methods:**

A cross-sectional study in 1027 patients with mild or moderate COVID-19 was performed in two communities in Bavaria, Germany. The Rehabilitation-Needs-Survey (*RehabNeS*) including the Short Form 36 Health Survey (*SF-36*) on health-related quality of life, was used. Descriptive statistics were calculated.

**Results:**

In all, 97.5% of patients reported one symptom in the infection stage, such as fatigue, respiratory problems, limitations of the senses of taste and smell, fear and anxiety and other symptoms. In this time period, 84.1% of the participants experienced activity limitations and participation restrictions such as carrying out daily routines, handling stress, getting household tasks done, caring for/supporting others, and relaxing and leisure concerns.

In all, 61.9% of participants reported persisting symptoms more than 3 months after infection. These were fatigue, sleep disturbances, respiratory problems, pain, fear and anxiety, and restrictions in movement; 49% of the participants reported activity limitations and participation restrictions. Predominately, these were handling stress, carrying out daily routines, looking after one’s health, relaxing and leisure activities and doing house work.

The impacts on quality of life and vocational performance were rather low.

**Conclusion:**

The results show that long-term symptoms after mild and moderate COVID-19 are common and lead to limitations of activities and participation. However, it seems that in most cases they are not severe and do not lead to frequent or serious issues with quality of life or work ability.

## Abstract (de)

**Hintergrund:**

Bei COVID-19-Überlebenden kann eine relativ hohe Anzahl von Langzeitsymptomen beobachtet werden. Neben der Beeinträchtigung der Lebensqualität können diese Symptome (jetzt als Post-COVID oder Long-COVID bezeichnet) auch Auswirkungen auf die Funktionsfähigkeit haben und die Betroffenen an der Teilnahme am sozialen Leben hindern. Es ist jedoch wenig bekannt, ob und in welchem Ausmaß Patienten und Patientinnen mit leichtem und mittelschwererem Verlauf, die keine intensivmedizinische oder normalstationäre Behandlung benötigten, ein solches Syndrom mit entsprechenden Defiziten entwickeln.

**Methode:**

In einer Querschnittsstudie wurden 1027 Patienten und Patientinnen mit leichtem oder mittelschwerem COVID-19 Verlauf in zwei Landkreisen in Bayern, fragebogenbasiert befragt. Hierzu wurde der Rehabilitation-Need-Survey (*RehabNeS)*, der den Short Form 36 Gesundheitsfragebogen (SF-36) zur gesundheitsbezogenen Lebensqualität beinhaltet, angewendet. Es erfolgte eine deskriptive Auswertung.

**Ergebnisse:**

97,5% der Patienten und Patientinnen berichteten über mindestens ein bestehendes Symptom in der Infektionsphase, wie Müdigkeit, Atemprobleme, Einschränkungen des Geschmacks- und Geruchssinns sowie Angst und Unruhe. In dieser Phase erlebten 84,1% der Befragten Aktivitätseinschränkungen und Einschränkungen der Teilhabe, z. B. in der Durchführung der täglichen Routine, der Bewältigung von Stress, der Erledigung von Aufgaben im Haushalt, die Pflege/Unterstützung anderer sowie bei der Fähigkeit sich zu entspannen.

61,9% der Teilnehmer und Teilnehmerinnen berichteten über anhaltende Symptome nach mehr als 3 Monaten nach der Infektion. Dies waren u.a. Müdigkeit, Schlafstörungen, Atemprobleme, Schmerzen, Ängste und Befürchtungen sowie Bewegungseinschränkungen. 49% der Befragten berichteten über Aktivitäts- und Teilnahmeeinschränkungen. Überwiegend waren dies der Umgang mit Stress, die Durchführung der täglichen Routine, die Pflege der Gesundheit, die Fähigkeit zur Entspannung und Freizeitaktivitäten sowie die Verrichtung von Hausarbeiten.

Die Auswirkungen auf die Lebensqualität und die berufliche Leistungsfähigkeit waren eher gering.

**Schlussfolgerung:**

Die Ergebnisse zeigen, dass Langzeitsymptome nach leichten und mittelschweren COVID-19 häufig sind und zu Einschränkungen von Aktivitäten und Teilhabe führen. Es scheint jedoch, dass sie in den meisten Fällen nicht sehr schwerwiegend sind und nicht zu häufigen oder schwerwiegenden Problemen in der Lebensqualität oder der beruflichen Leistungsfähigkeit führen.

## Introduction and background

Coronavrus infections are known to lead to Severe Acute Respiratory Syndrome (*SARS*) [1, 2]. When a novel Coronavirus has been detected in Wuhan, China, severe courses of the disease, leading to the need for intensive care, artificial respiration and/or Extra-Corporal-Membrane-Oxygenation (*ECMO*), have been observed [3]. More than 1 year after the first detection of Severe Acute Respiratory Syndrome Coronavirus Type 2 (*SARS-CoV-2*), more data about its severity and the course of infection are available. According to epidemiological data, Coronavirus Disease (*COVID-19*) leads to severe airway disease in around 15% of patients [3, 4], and around 5% of patients develop critical illness, mostly requiring artificial ventilation [4]. Around 80% of patients are asymptomatic or develop only mild or moderate symptoms [5]. Worldwide, around 2% of infected patients died from COVID-19-infections or related complications [6]. However, the reported death rates vary within wide ranges due to methodological differences. Risk factors for severe disease courses are old age, metabolic and renal disease, cardiovascular diseases, and obesity [7, 8].

Around 6 months after the description of COVID-19, Chinese researchers published observations that even weeks or months after the acute lung disease, long-term functional symptoms can be observed in a relatively high number of COVID-19 survivors [9]. Some of these symptoms can be seen as “non-specific”, others can be interpreted to result from infection or immune response in other organs or organ systems such as kidney, cardiovascular system, brain and the peripheral nervous system [9]. Frequently observed symptoms are fatigue, headache, dyspnoea and anosmia [10, 11]. Of course, pulmonary symptoms, such as coughing and dyspnoea and reduced cardiopulmonary performance, are also observed in patients with the so-called Post-COVID-Syndrome, frequently also called Long-COVID [12].

Besides impact on quality of life (*QoL*), Post-COVID-Syndrome has an impact on functioning and may hinder participation in social life in affected people. This may include being unfit for work, with impact on personal income and the productivity of society. For example, people with severe fatigue will not be able to work with machines, drive vehicles or do office work. If alterations of smell and taste occur, work in restaurants may not be possible any more, and alterations of motor functions may be a barrier for many jobs in the field of trade. These examples show that Post-COVID-Syndrome may be an indication for rehabilitation interventions [13]. This includes acute, post-acute and long-term rehabilitation [14–16].

Little is known if and to what extent patients with mild and moderate COVID-19 who did not need hospitalization or intensive care suffer from Post-COVID-Syndrome and how much QoL is affected. From the point of view of rehabilitation, an estimation of rehabilitation needs is of interest. In order to answer these questions a community survey in patients after mild and moderate SARS-CoV-2-infection in Germany was performed.

## Material and methods

For this cross-sectional study, a new written survey instrument was developed in cooperation be-tween the University Hospital Jena (*Institute for Physiotherapy*), the Hannover Medical School (*Department of Rehabilitation Medicine*) and the last author in her work for the Association of Statutory Health Insurance Physicians of Bavaria; a new written survey instrument was developed in early summer 2020. The Rehabilitation-Needs-Survey (*RehabNeS*) includes the established Short Form 36 Health Survey (*SF-36*) on health-related QoL and the newly created Rehabilitation-Needs-Questionnaire (*RehabNeQ*) with eleven dimensions and a total of 57 items that evaluates the rehabilitation needs of COVID-19 sufferers and additionally asks about satisfaction with the actors of the health care system and treatment in the context of infection [17].

After the positive vote of the Ethics Committee of the Medical Faculty of the Friedrich Schiller University Jena (*registration number 2020–1834-Bef*), contact was made with two Bavarian community public health departments. The contents of the questionnaire and aim of the study were explained and cooperation was agreed upon. Subsequently, a selection of the positively tested SARS-CoV-2 infected persons was carried out by the public health departments by the cut-off date 18. July 2020. Patients under 18 years of age, as well as residents of dementia homes, were excluded. A number of 1001 persons remained for whom the health departments requested the questionnaires. The study centre then sent the prepared envelopes containing the questionnaires to them. The local staff addressed and mailed the envelopes. There was no transmission of personal data to the study centre. In addition, 26 patients who had previously been in the direct care of the first author but lived in a different district were also involved and obtained the corresponding questionnaire with an anonymous return envelope. Patients did not receive a reminder letter. The costs of the material resources were covered by the participating universities.

The questionnaires were returned anonymized in a prepaid envelope to the Institute of Physiotherapy of Jena University Hospital. The data were processed in a descriptive way regarding the absolute and percentage frequencies. The patients were asked to specify their health problems on a scale of 1 to 5, where 1 means no problem and 5 means an extreme problem. With regard to the analysis presented here, the specifications were summarized as 2 to 5 being conspicuous. Furthermore, they should state if the problem still exists. The same questions were asked referring to the activity and participation of patients. Moreover, the SF-36 was evaluated using the official scoring system [18]. The calculated values are related to the results of the German norm sample.

## Results

### Sample characteristics

A total of 422 questionnaires were returned to the study center, representing a response rate of 41%.

**Fig. 1:**
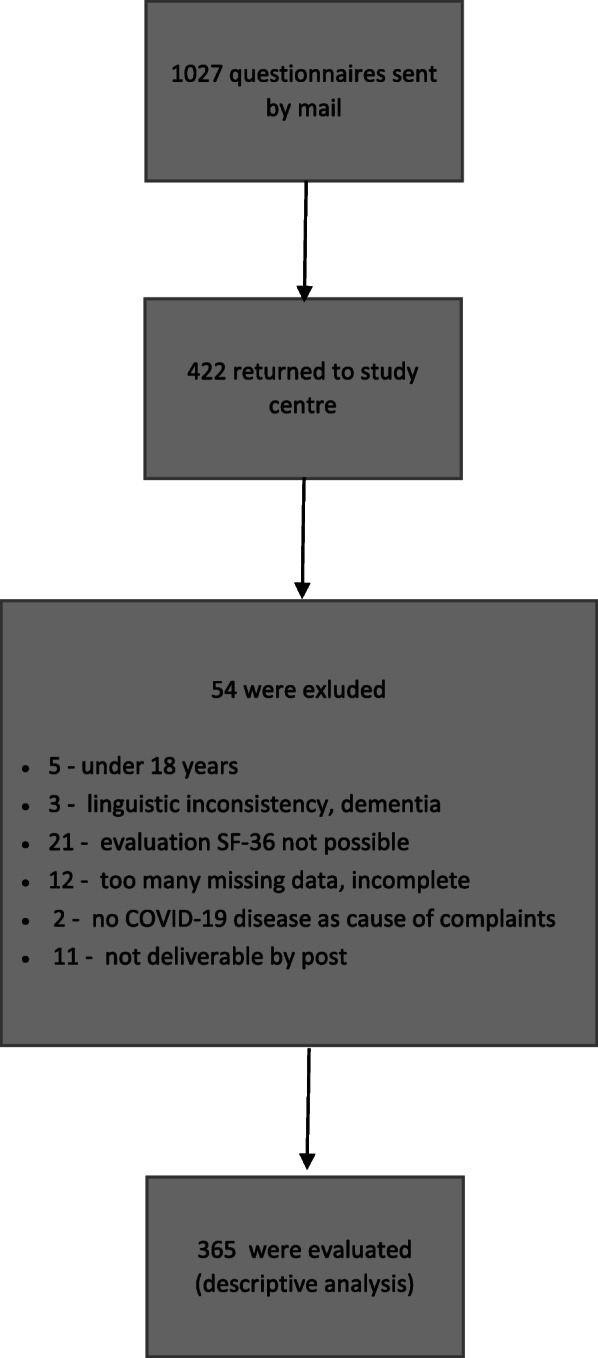
Flow chart of the study process

A total of 57 returns were excluded from the evaluation due to separate reasons (Fig. 1). For evaluation, 365 completely filled-in questionnaires were used; 216 (*59.2%*) of the respondents were female, and 148 (*40.5%*) were male. One respondent did not specify gender. The mean age of participants was 49.8 *(±16.9*) years, and 82% of the patients were within the age range of 18–64 years for remunerative employment (*or job training*) in Germany, which is common. The marital status, educational level and living situation were similar to the general German population. The majority of participants (*93.7%*) stated that the acute infection had been occurring more than 3 months before the survey.

### Activity and participation during the infection stage

In all, 84% of patients experienced activity limitations and participation restrictions in the infection phase.

**Fig. 2:**
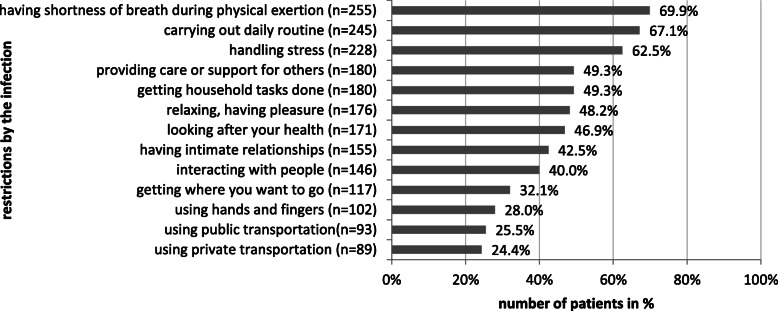
Problems in activity and participation during the infection stage

With regard to activities and participation during the infection stage the following problems were reported (Fig. 2):

- daily routine (*67.1%*)
- handling stress (*62.5%*)
- getting household tasks done (*49.3%*)
- caring for/supporting others (*49.3%*)
- relaxing, having pleasure (*48.2%*)
- looking after your health (*46.9%*)
- having intimate relationships (*42.5%*)
- interaction with people (*40.0%*)
- getting where you want to go (*32.1%*)
- using hands and fingers (*28%*)
- use of public transportation (*25.5%*)
- use of private transportation (*25.4%*)

### Symptoms and activity and participation at the time of survey

**Fig. 3:**
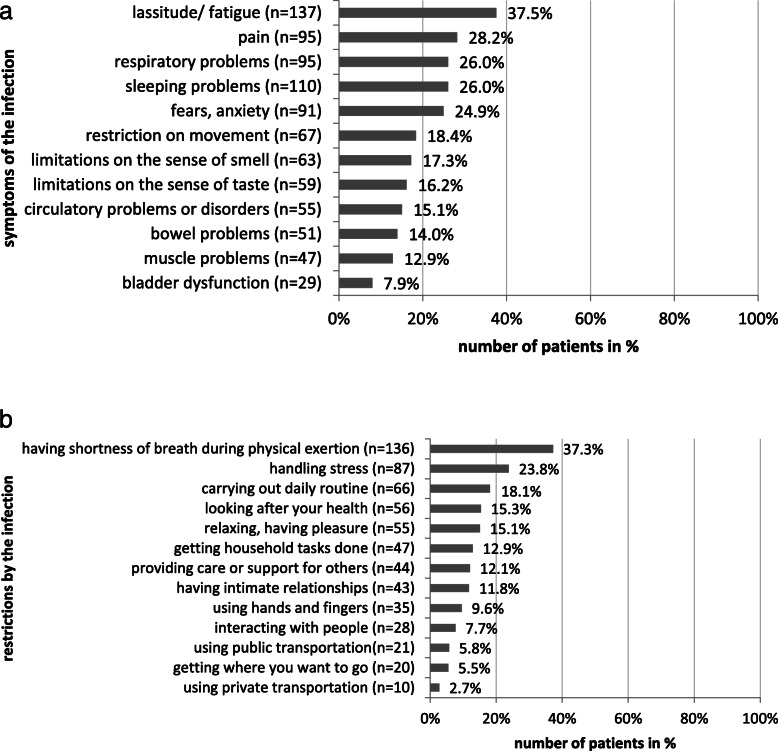
**a** Long-term Symptoms 3 months after infection**. b** Problems in activity and participation 3 months after infection

Summing up the number of symptoms, 226 participants (*61.9%*) of the total sample reported long-term symptoms. In 48 cases, one (*13.2%*); in 33 cases two (*9.0%*); in 33 more cases, three (*9.0%*); and in 112 cases, four or more symptoms (*30.7%*) were reported (Fig. 3a).

In all, 38.1% of cases did not report any long-term symptoms. The distribution of persisting symptoms was as follows: lassitude/fatigue (*37.5%*), sleep problems (*30.1%*), respiratory problems (*26.0%*), pain (*26.0%*), fear and anxiety (*24.9%*), restrictions on movement (*18.4%*), alterations of smell (*17.3%*) and taste (*16.2%*), cardiovascular problems (*15.1%*), bowel dysfunction (*14.0%*), muscular problems (*12.0%*), and bladder dysfunction (*7.9%*).

Summing up the number of reported problems in activity and participation in the long-term stage, 179 participants (*49.0%*) of the total sample reported long-term activity limitations and participation restrictions. In 56 cases, one (*15.3%*); in 23 cases, two (*6.3%*); in 24 cases three (6.6%); and in 76 cases, four or more (20.8%) problems were reported (Fig. 3b). The number of persisting problems after more than 3 months were:

- handling stress (*23.8%*)
- daily routine (*18.1%*)
- looking after your health (*15.3%*)
- relaxing and having pleasure (*15.1%*)
- getting household tasks done (*12.9%*)
- caring for/supporting others (*12.1%*)
- having intimate relationships (*11.8%*)
- using hands and fingers (*9.6%*)
- interactions with others (*7.7%*)
- use of public transportation (*5.8%*)
- getting where you want to go (*5.5%*)
- use of private transportation (*2.7%*)

According to physical exertion, 37.7% of patients still stated having shortness of breath.

### Quality of life

**Fig. 4:**
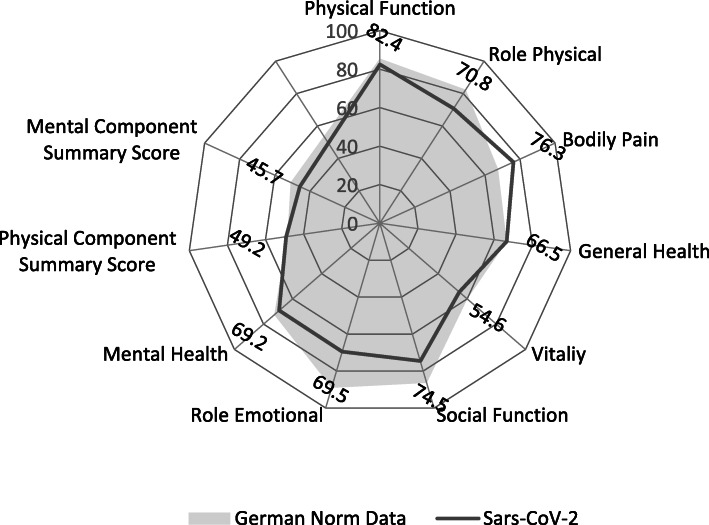
Results of SF-36 itemized by the single dimensions, entire study group in comparison to German norm data (*n = 365*)

In most cases the overall score of QoL was very good (*25.6%*) or good (*52.6%*). An average QoL was scored in 17.5% of patients. Bad (*3.9%*) or very bad (*0.3%*) QoL was stated in only a few cases. The mean values of the SF-36 questionnaire in the physical sum score was 49.2 points, which is in the range of the normal population (*48.4 points*) (Fig. 4). The Mental sum score was slightly reduced (*45.7* vs. *50.9 in normal population*). Reduced average scores were found in particular in the following dimensions Role physical (*70.8* vs. *82.4*), Vitality (*54.6* vs. *60.0*), Social function (*74.5* vs. *86.4*), Role emotional (*69.5* vs. *89.1*), and Mental health (*69.2* vs. *72.5*). In the subgroup of participants aged between 18 and 64 years, the values did not differ significantly (Table 1).

**Table 1:** Results of SF-36 (single dimensions and component scales) in participants in working age (18–64 years)

	PF	RP	BP	GH	VT	SF	RE	MH	PCS	MCS
18–64 years (n = 291)	86.7	76.8	78.2	68.7	55.6	76.5	74.5	70.4	50.8	46.3
In total (n = 365)	82.4	70.8	76.3	66.5	54.6	74.5	69.5	69.2	49.2	45.7

*PF* physical function, *RP* role physical, *BP* bodily pain, *GH* general health, *V* vitality, *SF* social function, *RE* role emotional, *MH* mental health, *PCS* physical component scale, *MCS* mental component scale

### Rehabilitation need/unfitness for work

Of the 291 participants aged 18–64 (*n = 291*), 255 (87.6%) participants declared to be in remunerative employment, 5 (1.7%) participants were seeking a job and 21 (*7.7%*) of the respondents did not have remunerative employment or received pension payments. In all, 2.4% of those who had a job had been classified by the doctors being unfit for work (*according to German social regulations*).

## Discussion

Overall, the results show that 3 months after mild and moderate COVID-19-disease, patients show at least one symptom in 61.9% of the cases. As these symptoms are thought to be related to the SARS-CoV-2 infection, they can be addressed as Post-COVID-Syndome [11, 19–21]. This percentage is similar to the findings by Jacobsen et al. [22] but clearly lower than that reported by Huang et al. [9]. The reason for this difference may be that Huang et al. had a higher number of severe and critical cases in their sample. Patients with longer artificial respiration periods and Intensive care treatment may develop SARS-CoV-2-independent symptoms, which have been described as Post-Intensive Care-Syndrome (*PICS*) (Flash MJ, Johnson SF, Nguemeni Tiako MJ, Tan-McGrory A, Betancourt JR, Lamas DJ, et al.: Disparities in post-intensive care syndrome during the COVID-19 pandemic: challenges and solutions, under review).

The symptom profile from our study demonstrates that, besides.

symptoms related to pneumonia, non-specific symptoms are predominant, such as fatigue, mental symptoms and pain. Additionally a number of symptoms may relate to alterations of the nervous system. This is congruent with findings from Wang et al. [23] and Lenzen-Schulte [21]. This profile shows similarities with the long-term symptoms of other severe diseases, such as cancer or auto-immune syndromes [24]. One explanation for these similarities may be that, in COVID-19 disease, after the primary lung infection, a second stage of the disease is observed. These symptoms can be explained by an overzealous-immune response [25]. However, the mechanisms of Post-COVID-Syndrome need further clarification.

Data on the impact of Post-COVID-Syndrome on physical functioning are rare. An Italian study described that about half of the Post-COVID-Syndrome patients had severe impairments in physical functioning and activities of daily living at hospital discharge [26]. Jacobson et al. [22] showed that 46% of the mildly affected patients and 73% of the hospitalized patients had an activity impairment due to the disease 3–4 months after their initial COVID-19 diagnosis. This is consistent with our findings that 49% of respondents reported at least one limitation of activities and/or restrictions in participation.

The results of the SF-36 showed only minor deviations in comparison with the normal population in Germany. Nevertheless, more than 4% of the respondents rated their current QoL as poor or very poor. This can be associated with major limitations from a personal perspective. An individual comparison of the QoL before and after the disease would be helpful for interpreting the limitation. But, from a methodological point of view, it is not possible in the context of the COVID-19 pandemic. The few deviations in the domains of the SF-36 in comparison to the normal population show that mild to moderate courses of the SARS-CoV-2 infection causes considerably less long-term alterations as compared with severe to critical progressions as well as other SARS diseases or acute respiratory distress syndrome [9, 27, 28]. Nevertheless, the relatively young population of this survey show some conspicuous results. At the level of sub scales, the SF-36 showed deficits in role physical, and slightly stronger deficits in social function and role emotional. This may be related to findings that mental disorders are frequently seen in patients after SARS-CoV-2 infection [11, 29]. The effects described are not significant when the group between 18 and 64 years is considered separately. However, the strongest deviations compared to the normal population also exist in the scales emotional role and social function. The long-term impact on activity and participation concerns only a minority of participants ranging from around 3 to 24%. The profile of alterations seems to relate to the above-mentioned non-specific symptoms and mental problems, within handling stress (*24%*), managing daily demands (*18%*) and problems with intimate relationships (*12%*) predominating.

The need for rehabilitation was not explicitly in the focus of the questionnaire used for the study. However, the observed symptoms, activity limitations and participation restrictions suggest that a relevant need for rehabilitation is existent in the population of individuals with mild and moderate SARS-CoV-2 infections. With regard to the symptoms, the percentages of persons who need rehabilitative interventions can be estimated at 15–35%. The detected impact on functioning results in a relatively lower percentage in need of rehabilitation (*estimated around 10–25%*). Work incapacity also occurs in the surveyed population, but its rate is relatively low (about 3%) compared to other results [30, 31]. This may have several reasons for its existence.. It might be a sign that people may compensate for the remaining problems relatively good after mild and moderate COVID-19. From the point of view of work performance, the percentage of people in need of rehabilitation may only be about 3%. Perhaps there was a certain bias in the vagueness of the formulated question. In the update of the questionnaire, we adjusted the expression of specific terms. The questionnaire is currently used regularly in the Post-COVID-Syndrome outpatient consultations of the Hannover Medical School (Clinic for Rehabilitation Medicine) and the University Hospital of Jena (Institute for Physiotherapy). Among the patients presenting here, the incapacity to work rate is more than 30%. However, this is a cohort that consults a doctor due to residual symptoms and cannot be fully compared to the sample described in this study.

The main **limitation** of the study is that it has been performed without a control group with matched samples. Due to urgency, it was not possible to recruit an appropriate control group. Recall and selection bias might be present. Due to the design of the questionnaire, respondents were also asked about symptoms and functional deficits that occurred at the time of infection. Looking back, this statement can lead to a bias in perception. However, it does not have an influence on the current functional deficits.

The response rate of 41% seems to be good in comparison to further surveys. However, the results must be interpreted due to response bias while being aware of the fact that possibly mainly affected persons answered who had even more restrictions or more symptoms [28, 29]. Furthermore, it may also be possible that those with the most severe functional impairments were unable to respond. Another possibility of response bias is that patients, who were already asymptomatic, did not respond (because they are maybe less interested). Hence there are maybe more affected patients in the study group. The predominance of female respondents can also be seen as a bias in the results, but other studies also showed more females with permanent symptoms after COVID-19. Under these circumstances, they are also more interested in reporting their existing symptoms [30, 32]. Another limitation is that we could not differentiate the severity of the SARS-COV-2 infection, because we had to use an anonymous data sampling approach. Also, no detailed information about the phenomenon of presentism at work place. It is worth considering if a more sensitive questionnaire of life could have made it possible to derive a more differentiated illustration.

In **conclusion**, this retrospective questionnaire-based survey shows that among patients with mild to moderate SARS-CoV-2 infection in the early stage of the disease, 84% of respondents reported activity and participation limitations, mainly in performing daily routines, coping with stress, household management, caring for / supporting others, and difficulties with leisure activities.

At the time of survey, infact 3 months after the acute infection, 61.9% of the participants reported at least one remaining symptom such as fatigue, sleep disturbances, respiratory problems, pain, fear respectively anxiety and movement restrictions. Almost half of the patients (*49%*) reported at least one activity limitation and participation restriction such as handling stress, carrying out daily routines, looking after own health, relaxing and leisure activities and carrying out house work.

Despite these high numbers of symptoms and activity restrictions, the overall QoL, as analysed with the SF-36 Health Survey, showed a relatively small reduction of mean values as compared to the German normal sample. This was also the case in the population of the working age population. Only a small group of patients with mild and moderate COVID-19 experience long-term unfitness for work.

These results show that long-term symptoms after mild and moderate COVID-19 are possible and lead to limitations of activities and participation. However, it seems that in most cases they are not very severe and do not lead to frequent or severe issues concerning QoL or work ability. Further investigations should be carried out here to detect the reasons and risks for long-term incapacity to work. The use of rehabilitative therapies should start at an early stage to enable a quick return to work. The high socio-economic impact on Post-COVID-Syndrome is a topic to be further developed.

## Data Availability

The datasets used and analysed during the current study are available from the corresponding author on reasonable request.
